# Extracellular Vesicles Derived From *Trichinella spiralis* Muscle Larvae Ameliorate TNBS-Induced Colitis in Mice

**DOI:** 10.3389/fimmu.2020.01174

**Published:** 2020-06-11

**Authors:** Yong Yang, Lei Liu, Xiaolei Liu, YuanYuan Zhang, Haining Shi, Wanzhong Jia, HongFei Zhu, Hong Jia, Mingyuan Liu, Xue Bai

**Affiliations:** ^1^Key Laboratory of Zoonosis Research, Ministry of Education, Institute of Zoonosis, College of Veterinary Medicine, Jilin University, Changchun, China; ^2^Mucosal Immunology and Biology Research Center, Massachusetts General Hospital, Boston, MA, United States; ^3^State Key Laboratory of Veterinary Etiological Biology, Key Laboratory of Veterinary Parasitology of Gansu Province, Lanzhou Veterinary Research Institute, Chinese Academy of Agricultural Sciences, Lanzhou, China; ^4^Institute of Animal Sciences, Chinese Academy of Agricultural Sciences, Beijing, China; ^5^Jiangsu Co-innovation Center for Prevention and Control of Important Animal Infectious Diseases and Zoonoses, Yangzhou, China

**Keywords:** *Trichinella spiralis*, extracellular vesicles, experimental colitis, immunomodulation, parasite-host communication

## Abstract

Helminths are masters at modulating the host immune response through a wide variety of versatile mechanisms. These complex strategies facilitate parasite survival in the host and can also be exploited to prevent chronic immune disorders by minimizing excessive inflammation. Extracellular vesicles (EVs) are small membrane-bound structures secreted by helminths which mediate immune evasion during parasite infection. The goal of this study was to investigate the immunoregulatory properties of *Trichinella spiralis* EVs (*Ts*-EVs) in a murine model of colitis. We found that *Ts*-EVs significantly ameliorated 2,4,6-trinitrobenzene sulfonic acid (TNBS)-induced colitis in mice. *Ts*-EVs alleviated intestinal epithelium barrier damage, markedly reduced pro-inflammatory cytokine secretion and neutrophil infiltration, and upregulated immunoregulatory cytokine expression in colon tissue. *Ts*-EVs also modulated the adaptive immune response by influencing T-cell composition. The numbers of Th1 and Th17 cells in MLNs, as well as the expression levels of Th1/Th17-associated cytokines and transcription factors in colon were reduced. In contrast, Th2 and Treg cells were increased after *Ts*-EVs treatment. Furthermore, sequencing of EV-derived microRNAs (miRNAs) indicated that an array of miRNAs was involved in the regulation of the host immune response, including inflammation. These findings expand our knowledge of host-parasite interactions, and may help design novel and effective strategies to prevent parasite infections or to treat inflammatory diseases like IBD. Further studies are needed to identify the specific cargo molecules carried by *Ts*-EVs and to clarify their roles during *T. spiralis* infection.

## Introduction

Trichinellosis is one of the most serious food-borne parasitic diseases in the world. It is caused by the intracellular parasitic helminth *Trichinella spiralis*, which is both a public health problem and a challenge for the economic productivity of the pig industry. Accidental ingestion of raw or undercooked meat containing infectious larvae of *T. spiralis* results in human infections. Due to changing diets and cooking habits, trichinellosis is considered an emerging or re-emerging infectious disease in several regions of the world (1).

In order to successfully invade and establish a chronic infection in the host and to prevent host immune attack, helminths, including *T. spiralis*, have evolved a variety of sophisticated immunoregulatory mechanisms. Some of these strategies have been identified, including induction of immune cell apoptosis, disruption of pattern recognition receptors (PRRs) and downstream signaling pathways, inhibition of the complement system, regulation of dendritic cell (DC) and macrophage differentiation toward tolerogenic DC and M2 macrophage phenotypes, expansion of the number of Treg cells and induction of Th2 immune responses (2). The ability of helminths to manipulate the host immune response and create an anti-inflammatory environment not only facilitate long-term parasite survival, but also protect against inflammatory disorders.

In this regard, growing numbers of epidemiological investigations in different regions of the world have found an inverse relationship between the prevalence of autoimmune diseases like IBD and parasitic infections, giving rise to the so-called “hygiene hypothesis” (3). Based on these investigations and hypothesis, the therapeutic potential of parasitic helminths against autoimmune diseases has been investigated in animal models. This approach has showed promising results, protecting against or alleviating numerous inflammatory conditions, such as inflammatory bowel disease (IBD), allergic airway inflammation, type I diabetes and autoimmune encephalomyelitis (4). Therefore, immunomodulatory “helminth therapy” is an attractive autoimmune therapy approach.

Diverse biomolecules released from or expressed on the surface of parasites, such as active enzymes and inhibitors, play a pivotal role mediating host-parasite interactions (5). Identification of parasite effector molecules has focused mainly on active protein moieties, whereas other functional structures have been neglected. Extracellular vesicles (EVs) are small membrane-bound vesicles secreted by parasites which contain functional proteins, carbohydrates, lipids, mRNA and non-coding RNAs. Recent studies showed that delivery of bioactive molecules and functional microRNAs from helminths to different host cells via EVs results in a series of intracellular signaling events that modulate host-parasite interactions and help parasites establish long term infections in inhospitable environments (6). For example, it was shown that EVs released by the gastrointestinal nematode *Heligmosomoides polygyrus* reduced Th2-type innate immune responses and eosinophilia induced by the allergenic fungus Alternaria, indicating that EVs can modulate the host innate immune response during parasitic infection (7). EVs released by *Echinococcus* metacestodes interfered with the antigen presentation pathway in murine dendritic cells and regulated macrophage activation through the LPS/TLR4 signaling pathway (8, 9). Using animal models of inflammatory diseases, studies have shown that EVs from *Nippostrongylus brasiliensis* and *Fasciola hepatica* can protect against inflammatory bowel disease (IBD) by regulating host immune balance (10, 11).

A recent study showed that *T. spiralis* muscle larvae also release EVs with immunomodulatory potential, with effects similar to whole ES L1 in regulating PBMC activity (12). Despite intense interest, information on the role of *T. spiralis* EVs in parasite-host communication is still limited. The goal of this study was to characterize, in detail, the miRNA composition of EVs released by the muscle larval stage of *T. spiralis*, and to investigate their potential regulatory effects on the host immune response in a murine model of colitis. Our results offer novel insights on the interaction between *T. spiralis* and the immune system of the host, on how this parasite immunoregulates its environment during infection, and also suggests new therapeutic tools to treat inflammatory diseases.

## Materials and Methods

### Animals and Ethics Statement

Female Balb/c mice aged 6–8 weeks (18–20 g) and female Wistar rats were purchased from the Experimental Animal Center of Jilin University. All animal experiments were in strict accordance with the National Institutes of Health guidelines (publication no. 85–23, revised 1996). Animals protocols were reviewed and approved by the Ethics Committee of Jilin University, affiliated with the Provincial Animal Health Committee, Jilin Province, China (Ethical Clearance number 201803022).

### Isolation of *T. spiralis* Muscle Larvae Extracellular Vesicles (*Ts*-EVs)

*Trichinella spiralis* (ISS534) was maintained in Wistar rats. Muscle larvae (ML) were recovered from the muscles of infected rats at 35 days post infection (dpi) by pepsin-HCl digestion, followed by cultivation in RPMI-1640 at 37°C and 5% CO_2_. After 18 h of culture, excretory/secretory products (ESPs) of ML were collected and centrifuged at 800 g/10 min and then at 5,000 g/20 min to remove parasite debris. The supernatant was filtered using low-protein binding 0.22 μm pore filters (Merck Millipore, USA) and then concentrated using a 10 kDa spin concentrator (Merck Millipore, USA) (13). For the isolation of *Ts*-EVs, concentrated ESPs were subjected to ultracentrifugation at 120,000 g for 2 h at 4°C using an Optima TL100 ultracentrifuge (Beckman Coulter, USA) with a TLA-55 rotor. They were washed with PBS and ultracentrifuged again at 120,000 g for 2 h at 4°C (14). The resulting pellets were resuspended in a small volume (200 μL) of PBS. *Ts*-EVs concentration was measured with a bicinchoninic acid (BCA) protein assay kit (Beyotime, China) and then *Ts*-EVs were stored at 4°C until use.

### Characterization of *Ts*-EVs by Transmission Electron Microscopy, NanoSight, and Western Blotting

Firstly, the morphology, structure and size of *Ts*-EVs were analyzed by negative staining transmission electron microscopy (TEM). Briefly, a 10 μL drop of *Ts*-EVs suspension was adsorbed onto a copper grid for 1 min and then immersed in 2% glutaraldehyde at room temperature. Then the grid was negatively stained using 2% phosphotungstic acid for 1 min, and air dried at room temperature. The *Ts*-EVs were examined using a HitachiH-7650 transmission electron microscope (Hitachi Limited, Japan) at 80 kV. Secondly, *Ts*-EVs particles were characterized in terms of their size and concentration using a NanoSight NS300 instrument (Malvern Instruments, United Kingdom).

Finally, to identify specific markers of *Ts*-EVs, equal amounts of samples (30 μg per well, *Ts*-EVs and ML crude proteins) were electrophoresed in a 12% SDS-PAGE gel and transferred to a PVDF membrane (Immobilon, Millipore, USA). The membrane was blocked with 5% bovine serum albumin (BSA) and incubated with the primary antibody, which included polyclonal rabbit anti-CD63 (1:1,000, Abcam, United Kingdom) and polyclonal goat anti-enolase (1:200, Abcam, United Kingdom). Subsequently, two different horseradish peroxidase (HRP)-conjugated secondary antibodies were used: goat anti-rabbit IgG (1:2,000, Cell Signaling, USA) and donkey anti-goat IgG (1:50,000, Jackson ImmunoResearch, USA). Peroxidase activity was visualized using the ECL Plus Western blotting detection system (GE Healthcare Buckinghamshire, UK).

### TNBS-Induced Colitis and Treatment With *Ts*-EVs

Colitis was induced by intrarectal administration of 100 μL of 1.25 mg 2,4,6-trinitrobenzene sulfonic acid (TNBS) solution (Sigma, USA). A total number of 40 mice were randomly assigned to 4 different groups (10 mice/group): PBS as control group; TNBS group; TNBS+PBS group; and TNBS+*Ts*-EVs group. Briefly, mice deprived of food for 24 h were lightly anesthetized with sodium pentobarbital (50 mg/kg, ip). Next, colitis was induced by intrarectal administration of TNBS (1.25 mg in 100 μL of a 50% ethanol solution) through a flexible catheter inserted 3.5 cm into the rectum. Before the induction of colitis, mice in the TNBS+*Ts*-EVs group were injected intraperitoneally three times with *Ts*-EVs (50 μg/mice) resuspended in 100 μL PBS, and mice in the TNBS+PBS group were injected intraperitoneally (IP) three times with PBS as control group. All mice were sacrificed 3 days after intrarectal administration of TNBS.

### Assessment of Colitis

Mice were observed daily and scored for disease severity by using a 6-point scale disease activity index (DAI). Scores depended on weight loss, stool shape, and presence of blood in the stool (Table 1). On day 3, all mice were killed and their colons were removed. We measured the length of the colon as an indirect indicator of inflammation and macroscopically assessed the degree of colonic damage. Three parameters were considered: degree of hyperemia, wall thickness, and degree of colonic ulceration. The total score ranged from 0 to 9 (Table 2). All scores were determined by observers blinded to the treatment groups.

**Table 1:** The criteria of Disease Activity Index (DAI) score.

**Weight loss (%)**	**Stool shape**	**Stool bleeding**	**Score**
<4%	Normal	None	0
4-10%	Loose stool	Slight bleeding	1
>10%	Diarrhea	Severe bleeding	2

**Table 2:** The criteria of colonic macroscopic score.

**Hyperemia**	**Wall thickening**	**Ulcer**	**Score**
None	None	None ulcer	0
Focal	Slight	Slight ulcer	1
Multifocal	Moderate	Moderate ulcer	2
Diffuse	Severe	Severe ulcer	3

Approximately 1 cm of colon was resected for histopathology examination, fixed in 4% neutral-buffered formalin, embedded in paraffin, sectioned at 5 μm thickness and stained separately with hematoxylin and eosin (H&E) and Periodic Acid-Schiff (PAS). We determined the histological damage score to measure the severity of inflammation based on the following parameters (Table 3): extent of inflammation, inflammatory cell infiltration, extent of crypt damage, and loss of goblet cells. The total score ranged from 0 to 8.

**Table 3:** The criteria of colonic histopathological score.

**Extent of inflammation**	**Inflammatory cell infiltration**	**Extent of crypt damage**	**Loss of goblet cells**	**Score**
None	None	None	None	0
Mucous layer	Focal	Slight	Focal	1
Serous layer	Diffuse	Severe	Diffuse	2

### MPO Activity Assay

Inflammatory cell (polymorphonuclear neutrophil) infiltration into colonic tissue was quantified by measuring MPO activity with an MPO assay kit (Nanjing Jiancheng Bio-engineering Institute, China), following the manufacturer's instructions. MPO activity was expressed as units per gram of total protein (U/g).

### Intestinal Permeability Assay

Intestinal permeability was assessed by using fluorescein isothiocyanate-labeled dextran (FITC-D) with molecular weight of 4,000 Da (Sigma). Briefly, 2 days after induction of colitis by TNBS treatment, mice were orally administrated with 600 mg/kg of FITC-D. After 4 h administration, blood was collected from the eyes of the mice and centrifuged at 12,000 g for 5 min to separate serum. Fluorescence intensity in the serum was measured by a microplate reader with excitation and emission wavelengths of 490 and 525 nm, respectively. The concentrations of FITC-D were calculated by a standard curve generated by serial dilution of FITC-D. Each sample was measured in triplicate.

### Immunofluorescence Analysis

Colonic tissue sections were deparaffinized and subjected to an antigen retrieval process. Briefly, sections were heated in sodium citrate buffer (pH 6.0) with a 500-watt microwave oven until the temperature reached 100°C. When the temperature of the sodium citrate buffer dropped below 60°C, tissue slices were treated with protease K. Non-specific binding was blocked by incubating with 5% normal goat serum for 30 min at room temperature. For occludin and zonula occludens-1 (ZO-1) staining, sections were separately incubated with rabbit monoclonal anti-occludin antibody (1:50 dilution; Abcam, USA) and rabbit polyclonal anti-ZO-1 antibody (1:200 dilution; Abcam, USA) overnight at 4°C. After wards, sections were washed with PBS and incubated with Alexa Fluor 555 goat anti-rabbit IgG antibody (1:400 dilution; Abcam, USA) in the dark for 45 min at room temperature. Sections were then washed with PBS, stained with Hoechst dye (1:2,000 dilution; Abcam, USA) for 5 min and washed again with PBS. Finally, images of the stained sections were analyzed by confocal microscopy.

### Cytokine Assays

Colon tissue was homogenized in tissue protein extraction reagent (Thermo Scientific, USA). After completion of lysis, the homogenized tissue was centrifuged at 12,000 g for 10 min. The collected supernatant was stored at −80°C until analysis. Levels of Th1 cytokines (IL-1β, IFN-γ, and TNF-α), Th2 cytokines (IL-4 and IL-13), a Th17 cytokine (IL-17A), regulatory cytokines (IL-10 and TGF-β) and chemokines (MCP-1 and MIP-3α) were analyzed using the Meso Scale Discovery (MSD) electrochemiluminescence platform. All measurements were performed in triplicate. The average absorbance at 620 nm was determined for each sample and was used to calculate cytokine concentrations in picograms per milliliter (pg/mL).

### RNA Extraction and Quantitative Real-Time PCR

Colon RNA was extracted, purified (Qiagen, Germany) and converted to cDNA (Stratagene, USA), following the manufacturer's instructions. Quantitative real-time PCR was performed using FastStart Universal SYBR Green Master (Rox) reagents (Roche Diagnostics, Indianapolis, IN) and a 7500 Real-Time PCR machine (Applied Biosystems, Foster City, CA). Primer sequences are listed in Table 4. The reaction conditions were: 95°C for 10 min; followed by 40 cycles of 95°C for 15 s, 56°C for 1 min and 72°C for 1 min; and concluding with a melting curve analysis. Fold change in gene expression was calculated using the 2^−ΔΔCt^ method.

**Table 4:** Primers used for real-time PCR analysis.

**Genes**	**Primer**	**Sequence(5′-3′)**
T-bet	Forward primer	TCAACCAGCACCAGACAGAG
	Reverse primer	AACATCCTGTAATGGCTTGTG
RORγt	Forward primer	AGTGTAATGTGGCCTACTCCT
	Reverse primer	GCTGCTGTTGCAGTTGTTTCT
GAGT3	Forward primer	CTTATCAAGCCCAAGCGAAG
	Reverse primer	CCCATTAGCGTTCCTCCTC
Foxp-3	Forward primer	GGTATATGCTCCCGGCAACT
	Reverse primer	GATCATGGCTGGGTTGTC
IFN-γ	Forward primer	GCTCTGAGACAATGAACGCT
	Reverse primer	AAAGAGATAATCTGGCTCTGC
IL-17A	Forward primer	ATCCCTCAAAGCTCAGCGTGTC
	Reverse primer	GGGTCTTCATTGCGGTGGAGAG
IL-4	Forward primer	TTGTCATCCTGCTCTTCTTTCT
	Reverse primer	CTGTGGTGTTCTTCGTTGCT
IL-10	Forward primer	CCTCAGTTCCCATTCTATTTATTCACT
	Reverse primer	TTGAAAGGACACCATAGCAAAGG

### Flow Cytometry Analysis

All antibodies and reagents were purchased from BD (BD Biosciences, USA). Mesenteric lymph node (MLN) cells were isolated from different groups of mice. For analysis of intracellular cytokine expression, MLN cells were stimulated with Leukocyte Activation Cocktail (2 μL of Cocktail for every 1 mL of cell culture; approximately 1 × 10^6^ cells/mL) containing PMA, ionomycin and GolgiPlug™ (Brefeldin A) for 5 h at 37°C and 5% CO_2_. Next, the cultured cells were collected and incubated with purified monoclonal rat anti-mouse CD16/CD32 (0.5 mg/mL) antibody for 10 min to block non-specific staining, and then with FITC-conjugated monoclonal rat anti-mouse CD4 (0.5 mg/mL) antibody for 30 min at 4°C. After fixation and permeabilization with Cytofix/Cytoperm solution, the cells were intracellularly stained with APC-conjugated monoclonal rat anti-mouse IFN-γ and IL-4 (0.2 mg/mL) antibody, PE-conjugated monoclonal rat anti-mouse IL-13 (eBioscience, USA) and IL-17A (0.2 mg/mL) antibody for 45 min at 4°C. In order to analyze CD4^+^ CD25^+^ Foxp3^+^ Treg cells, MLN cells were first incubated with FITC-conjugated monoclonal rat anti-mouse CD4 (0.5 mg/mL) antibody and APC-conjugated monoclonal rat anti-mouse CD25 (0.2 mg/mL) antibody for 30 min at 4°C. After washing, cells were fixed, permeabilized and stained with PE-conjugated monoclonal rat-anti mouse Foxp3 (0.2 mg/mL) antibody for 30 min at 4°C. The corresponding fluorochrome-labeled isotype control antibodies served as negative controls. Data were acquired on a FACS Aria II flow cytometer (BD Biosciences) and samples were analyzed using FlowJo software (TreeStar, USA).

### Small RNA Library Construction and Bioinformatics Analysis

Biological replicates of *T. spiralis* EVs (*Ts*-EVs) isolated from three different batches of muscle larvae were analyzed. For the analysis of small RNAs, total RNA from *Ts*-EVs was extracted using Trizol according to the manufacturer's instructions (Life Technologies). Purity and concentration of total RNA were analyzed with an Agilent 2100 system (Agilent Technologies, Santa Clara, CA, USA) to make sure that samples were of sufficiently high quality for sequencing. Small RNAs between 18 and 30 nt were isolated by 15% polyacrylamide gel electrophoresis (PAGE) and small RNA libraries were prepared using a TruSeq Small RNA Library Prep Kit (Illumina, San Diego, CA, USA), following the manufacturer's instructions. The generated libraries were sequenced using an Illumina^®^HiSeq 2000/2500 system (Beijing Genomics Institute, Shenzhen, China).

After filtering the raw data, the clean reads were mapped to the draft genome sequence of *T. spiralis* using SOAP software. For miRNA analysis, miRBase v. 22 and GenBank was used to identify known *T. spiralis* miRNAs from the clean reads. Finally, the unmatched small RNAs were analyzed using Mireap software (http://sourcefor-ge.net/projects/mireap) for novel miRNA prediction analysis based on the hairpin structure characteristics of the miRNA precursor. Only common overlapping miRNA sequences in all replicates were selected for further analyses. To determine the potential roles of specific miRNAs from *Ts*-EVs in host cells, miRanda and TargetScan were used to predict the mouse target genes of miRNAs. The predictions from the two tools were combined and the common target genes were the basis for further analysis. A clustering heatmap was drawn using the statistical programming language R package pheatmap (http://cran.r-project.org/web/packages/pheatmap/index.html) and the ggplot2 package was utilized to identify and annotate the target genes.

### Statistical Analysis

Results are expressed as the mean ± standard deviation (SD). Statistical analysis was performed using the GraphPad Prism 5 software for Windows. One-way analyses of variance (ANOVA) followed by Tukey's multiple-comparison test were used to compare significant differences between different conditions. Different *p*-values are shown as ^*^p < 0.05, ^**^p < 0.01 and ^***^p < 0.001.

## Results

### Characterization of *T. spiralis* Muscle Larvae Extracellular Vesicles (*Ts*-EVs)

**Figure 1:**
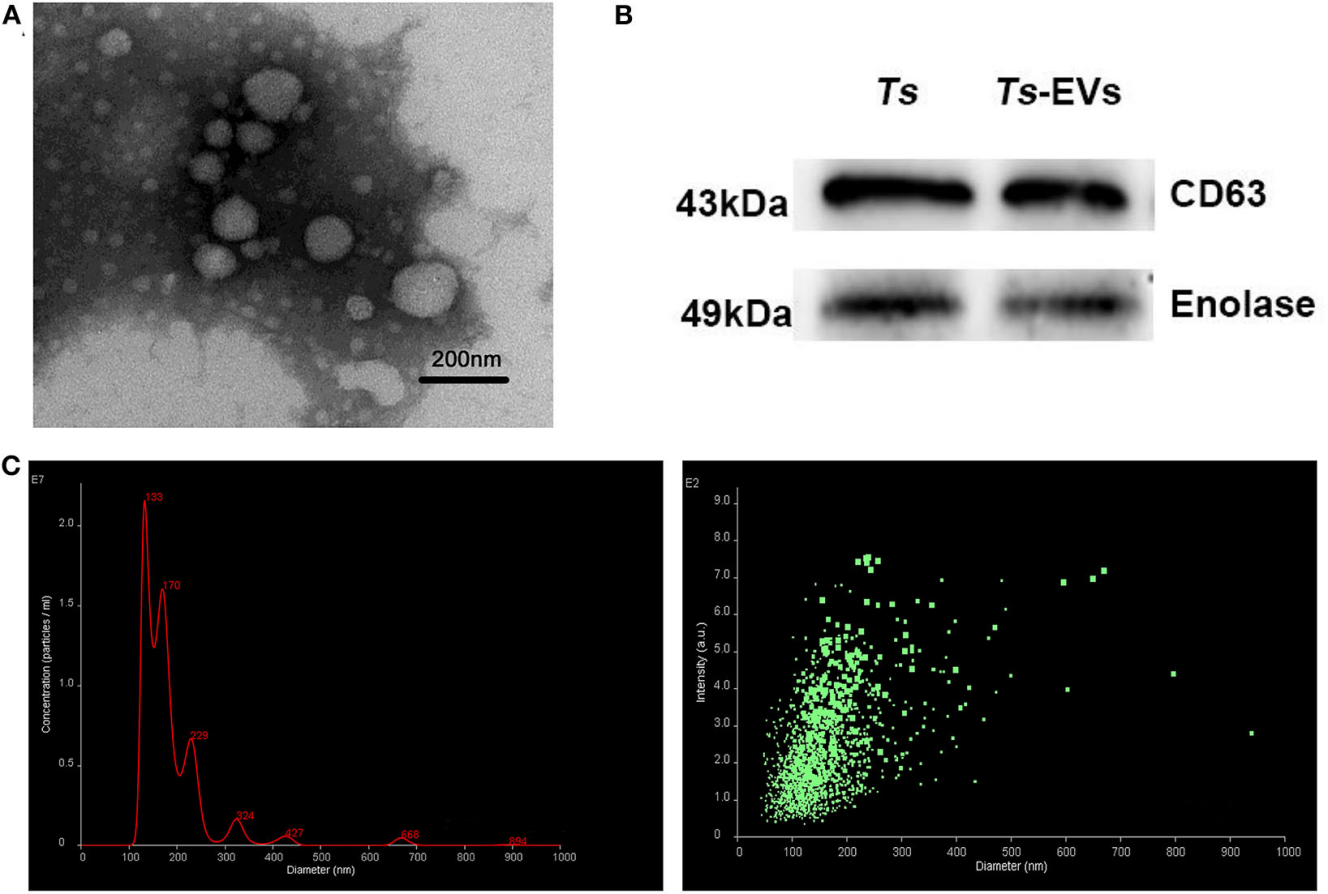
Characterization of *T. spiralis* muscle larvae extracellular vesicles (*Ts*-EVs). **(A)** *Ts*-EVs ultrastructure was visualized by negative-staining TEM (magnification 40,000×; scale-bars = 200 nm). **(B)** Expression of *Ts*-EVs-specific markers CD63 and enolase was determined by Western blotting. **(C)** Size distribution profile of *Ts*-EVs was investigated using NTA. TEM: Transmission electron microscopy; NTA: Nano tracking analysis.

In order to verify the quality of the *Ts*-EVs preparations, transmission electron microscopy (TEM) was used to evaluate their size and morphology. TEM revealed that most EVs were closed round vesicles with a diameter of 30–150 nm (Figure 1A). Western blot analysis showed that the specific markers of EVs, CD63 and enolase, were present in *Ts*-EVs (Figure 1B). Furthermore, NanoSight was used to study the size distribution of EVs. This analysis showed that the peak size of *Ts*-EVs was 133 nm and that most measured between 50 and 250 nm (Figure 1C). All these data indicate that these vesicles are *Ts*-EVs.

### *Ts*-EVs Ameliorate the Severity of TNBS-Induced Colitis in Mice

**Figure 2:**
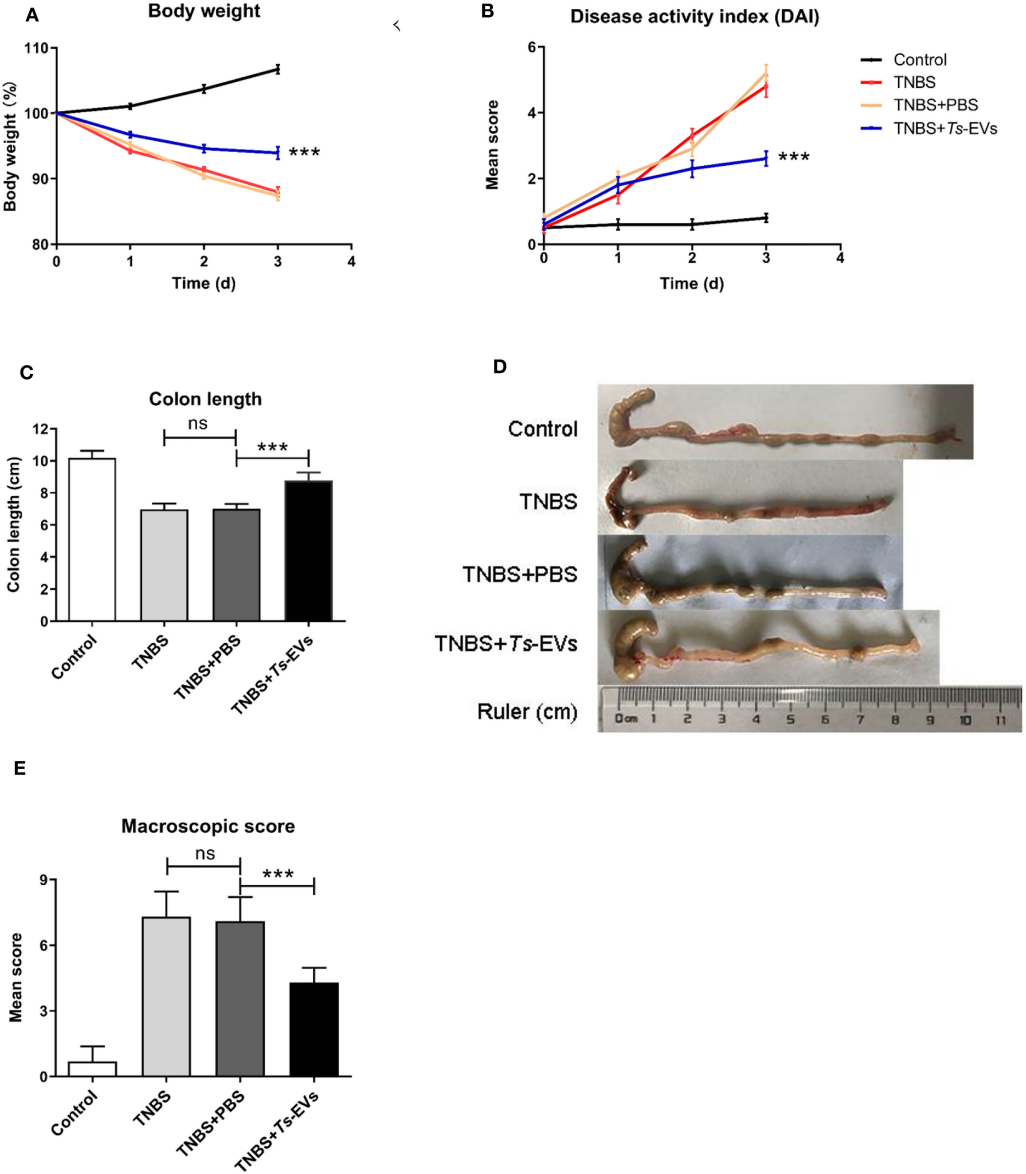
*Ts*-EVs ameliorate clinical symptoms and preserve the normal macroscopic structure and length of the colon in TNBS-induced colitis. Mice received three intraperitoneal injections of *Ts*-EVs (50 μg) prior to intrarectal administration of TNBS. The protective effect against TNBS-induced colitis was determined in three independent experiments. One representative experiment is shown here. **(A)** Body weight was recorded daily in each group. **(B)** The disease activity index (DAI) was evaluated daily based on three parameters (weight loss, stool shape, and presence of blood in stool), as described in methods (day 3, ^***^p < 0.001, TNBS + PBS vs. TNBS + *Ts*-EVs). **(C,D)** After 3 days, colons were removed and their length was measured as an indirect marker of inflammation. **(E)** Mean macroscopic scores of colonic inflammation based on 3 parameters (hyperemia, wall thickening, and ulcers), as described in methods. Results are shown as mean ± standard deviations (SD) of each group (*n* = 10 per group). Statistical analysis was performed with one-way ANOVA followed by Tukey's multiple-comparison test. ^***^p < 0.001.

To test whether *Ts*-EVs could ameliorate TNBS-induced colitis, Balb/c mice were intraperitoneally injected three times with *Ts*-EVs (50 μg/mice) every 3 days before colitis induction. As expected, intrarectal TNBS administration to mice induced obvious symptoms of colitis such as weight loss (13%), diarrhea and rectal bleeding, compared with the control mice. However, the rate of body weight loss in the TNBS+*Ts*-EVs group was significantly reduced on the final day of the experiment (Figure 2A, *p* < 0.001). The DAI scores in the TNBS group, assessed according to Table 1, were dramatically increased (Figure 2B). Mice in the TNBS group typically showed reduced colon length, whereas the colon length in the TNBS+*Ts*-EVs group was significantly increased (Figures 2C,D, *p* < 0.001). In addition, colon macroscopic scores, evaluated based on Table 2, showed that mice treated with *Ts*-EVs prior to TNBS induction displayed markedly suppressed macroscopic signs of colonic involvement when compared with the TNBS group (Figure 2E, *p* < 0.001). There were no significant differences between the TNBS and TNBS+PBS groups.

**Figure 3:**
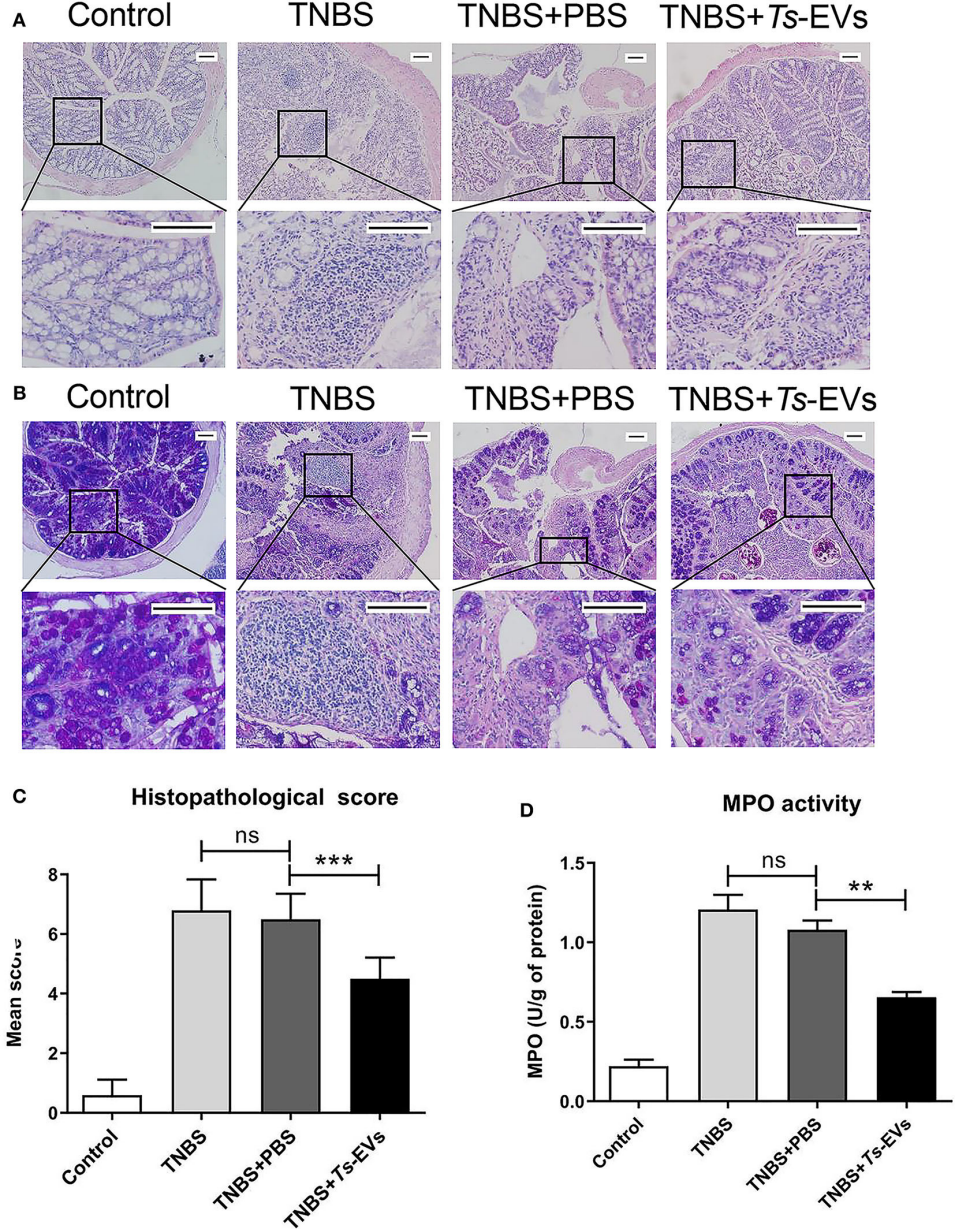
*Ts*-EVs reduced histopathological signs of colon damage and myeloperoxidase (MPO) activity in TNBS-induced colitis. **(A)** Colon tissue samples were examined after hematoxylin and eosin (H&E) staining (magnification 100× and 400×, scale-bars = 100 μm). **(B)** Colon tissue samples were examined after periodic acid Schiff (PAS) staining (magnification 100× and 400×, scale-bars = 100 μm). **(C)** Histopathological scores based on the extent of inflammation, inflammatory cell infiltration, extent of crypt damage and loss of goblet cells, as described in methods. **(D)** Myeloperoxidase (MPO) activity was determined by a spectrophotometric method. Results are shown as mean ± SD of each group (*n* = 10). Statistical analysis was performed with one-way ANOVA followed by Tukey's multiple-comparison test. ^**^p < 0.01, ^***^p < 0.001.

We also analyzed histopathological changes in the colon by H&E and PAS staining. The colonic mucosa was clearly damaged in the TNBS group, showing epithelial destruction, intense inflammatory infiltration, significant crypt damage and loss of goblet cells. In contrast, administration of *Ts*-EVs prior to colitis induction prevented damage of epithelial structures and reduced inflammatory infiltration (Figure 3A). Interestingly, PAS staining of colon tissue showed widespread reduction of goblet cells in mice with TNBS-induced colitis, whereas the goblet cells in the TNBS+*Ts*-EVs group showed only focal areas of reduction (Figure 3B). This is consistent with previous H&E stainings examining goblet cells. We evaluated the H&E and PAS staining results based on the criteria listed in Table 3 and concluded that *Ts*-EVs administration clearly reduced the degree of histological damage when compared with the TNBS group (Figure 3C, *p* < 0.001). MPO activity reflects the degree of infiltration of neutrophils into various tissues. Our results showed that *Ts*-EVs treatment significantly reduced colonic MPO activity when compared with the TNBS group (Figure 3D, *p* < 0.001). Altogether, these results demonstrate that *Ts*-EVs can ameliorate the severity of TNBS-induced colitis.

### *Ts*-EVs Reduce the Permeability and Enhance the Expression Levels of Occludin and ZO-1 Tight Junction Protein in TNBS-Induced Colitis

**Figure 4:**
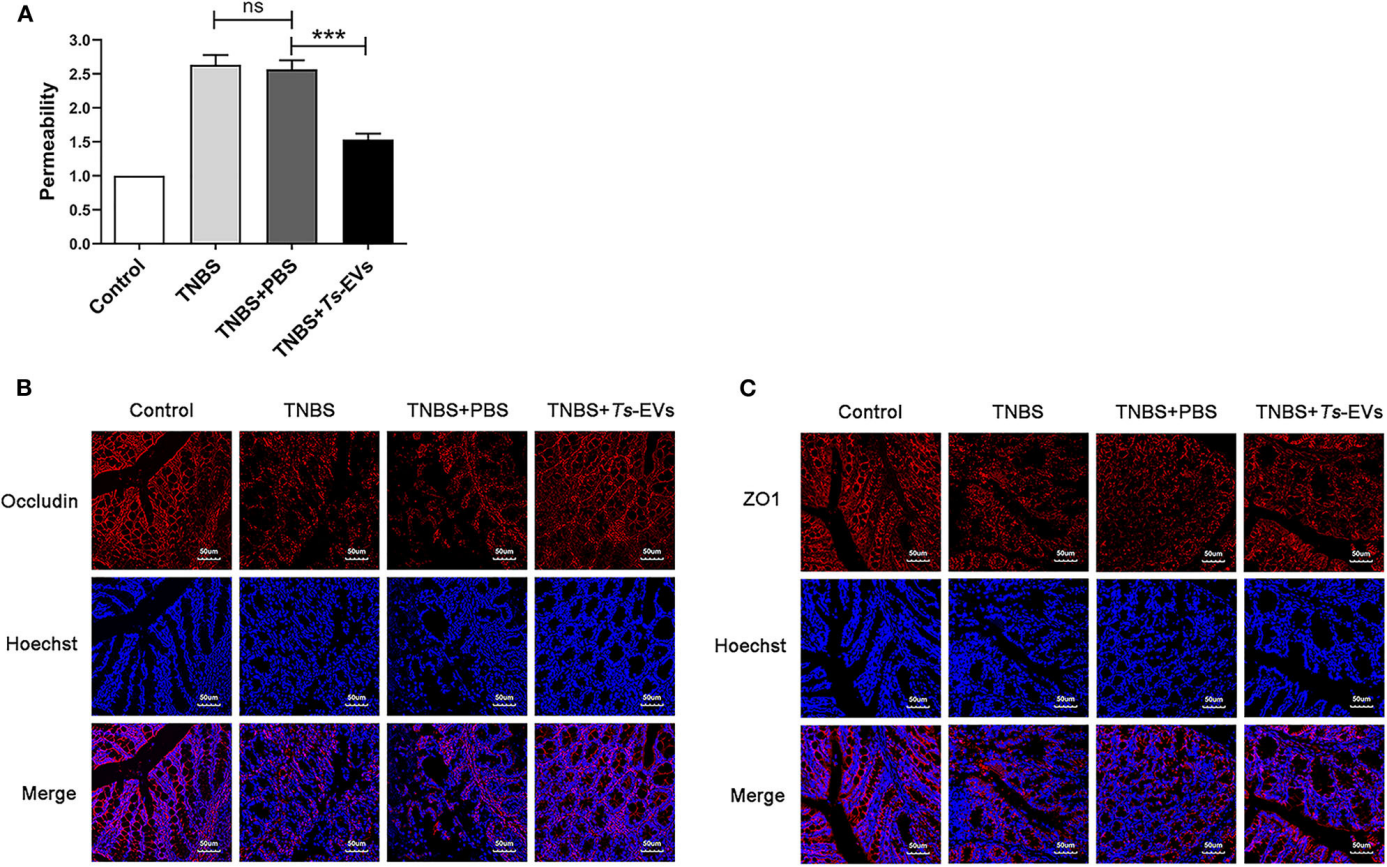
*Ts*-EVs restored the impaired permeability and preserved tight junctions integrity inTNBS-induced colitis. **(A)** Intestinal permeability was measured by FITC-Dextran permeability assay. Relative permeability of TNBS and *Ts*-EV treatment in mice. Results are shown as mean ± SD of each group (*n* = 3). Statistical analysis was performed with one-way ANOVA followed by Tukey's multiple-comparison test. ^***^p < 0.001. **(B,C)** Tight junction proteins were measured by using laser scanning confocal microscopy. **(B)** Colonic occludin (red), nuclei (blue). **(C)** Colonic ZO-1 (red), nuclei (blue). Magnification of the images was 400×, Scale-bars = 50 μm. These figures are representative of three independent experiments.

To explore the effects of *Ts*-EVs on intestinal barrier integrity during TNBS-induced acute colitis, we assessed intestinal permeability using FITC-D. As shown in Figure 4A, TNBS administration significantly increased intestinal permeability compared with the control group. In contrast, preventive treatment with *Ts*-EVs effectively counteracted the TNBS-induced increase in intestinal permeability in mice. In addtion, we examined the expression levels of occludin and ZO-1 protein in colonic tissues by immunofluorescence. Occludin and ZO-1 are located on the membrane of colonic mucosal epithelial cells and play a crucial role protecting the integrity of the intestinal mucosal barrier and regulating intestinal permeability. Immunofluorescence staining showed that the levels of expression of occludin in the TNBS+*Ts*-EVs group were significantly higher than in the TNBS group (Figure 4B). In addition, weaker ZO-1-specific fluorescence was observed in the TNBS group, whereas the expression of ZO-1 was significantly enhanced in the *Ts*-EVs-treated group (Figure 4C).

### *Ts*-EVs Regulate the Expression Levels of Inflammatory Cytokines in TNBS-Induced Colitis

**Figure 5:**
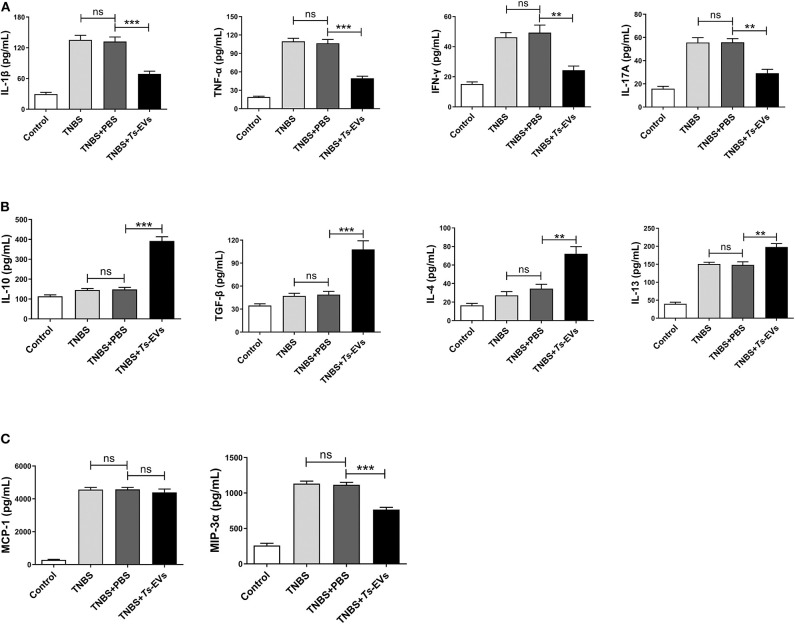
Levels of cytokines and chemokines in colons of mice with TNBS-induced colitis pretreated or not with *Ts*-EVs. Cytokines levels in homogenized colon tissue supernatants were determined by MSD. **(A)** Expression levels of Th1 cytokines (IL-1β, IFN-γ, and TNF-α) and a Th17 cytokine (IL-17A). **(B)** Expression levels of Th2 cytokines (IL-4 and IL-13) and regulatory cytokines (IL-10 and TGF-β). **(C)** Expression levels of chemokines (MCP-1 and MIP-3α). Results are shown as mean ± SD of each group (*n* = 3). Statistical analysis was performed with one-way ANOVA followed by Tukey's multiple-comparison test. ^**^p < 0.01, ^***^p < 0.001.

Inflammatory cytokines play a central role in the pathogenesis of IBD. To determine whether the protective effects of *Ts*-EVs against TNBS-induced colitis in mice were associated with inhibition of inflammatory cytokines, supernatants from homogenized colonic tissue were collected. The levels of Th1 or pro-inflammatory cytokines (IL-1β, IFN-γ, and TNF-α), Th2 cytokines (IL-4 and IL-13), Th17 cytokine (IL-17A), anti-inflammatory cytokines (IL-10 and TGF-β) and chemokines (MCP-1 and MIP-3α) were analyzed by MSD. As shown in Figure 5, TNBS administration induced the release of all the aforementioned inflammatory cytokines compared with the control group. In contrast, preventive treatment with *Ts*-EVs significantly decreased the expression levels of the pro-inflammatory cytokines IL-1β (*p* < 0.001), TNF-α (*p* < 0.001), IFN-γ (*p* < 0.01), and IL-17A (*p* < 0.01) when compared with the TNBS group (Figure 5A). Moreover, compared with the TNBS group, the expression levels of the anti-inflammatory cytokines IL-10 (*p* < 0.001) and TGF-β (*p* < 0.001), and of the Th2-related cytokines IL-4 (*p* < 0.01) and IL-13 (*p* < 0.01), were significantly higher in mice treated with *Ts*-EVs (Figure 5B). Interestingly, compared with the TNBS group, there was no significant change in chemokine MCP-1 expression after *Ts*-EVs treatment, but the levels of chemokine MIP-3α (*p* < 0.001) were significantly reduced (Figure 5C). Collectively, our findings indicate that *Ts*-EVs inhibit the production of pro-inflammatory cytokines and upregulate the levels of anti-inflammatory cytokines, contributing to the inhibition of colitis.

### *Ts*-EVs Regulate T Cell Differentiation During TNBS-Induced Colitis

**Figure 6:**
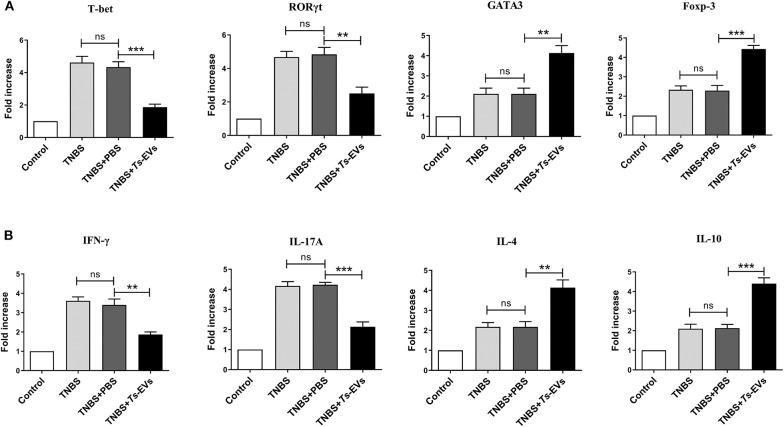
qReal-time PCR analyses of gene expression levels in colons of mice with TNBS-induced colitis pretreated or not with *Ts*-EVs. **(A)** T-bet, GATA3, RORγt and Foxp-3 mRNA expression levels in the colons were measured by qRT-PCR. **(B)** IFN-γ, IL-17A, IL-4, and IL-10 mRNA expression levels. Results are shown as mean ± SD of each group (*n* = 3). Statistical analysis was performed with one-way ANOVA followed by Tukey's multiple-comparison test. ^**^p < 0.01, ^***^p < 0.001.

To explore the effects of *Ts*-EVs on the local T-cell immune response during TNBS-induced colitis, the levels of mRNA coding for T helper- and Treg-associated cytokines such as IFN-γ, IL-4, IL-17A, IL-10, as well as for the transcription factors T-bet, GATA-3, ROR-γt, Foxp3 were analyzed by qRT-PCR after extracting total mRNA from colon tissue. Compared with the TNBS group, the TNBS+*Ts*-EVs group showed significant inhibition of T-bet (*p* < 0.001) and RORγt (*p* < 0.01) mRNA expression, while the expression levels of GATA3 (*p* < 0.01) and Foxp3 (*p* < 0.001) mRNA were increased (Figure 6A). Consistent with the levels of mRNA coding for their respective transcription factors, the qRT-PCR analysis showed that IFN-γ (*p* < 0.01) and IL-17A mRNAs (*p* < 0.001) were significantly reduced, whereas IL-4 (*p* < 0.01) and IL-10 mRNA (*p* < 0.001) expression was upregulated by treatment with *Ts*-EVs (Figure 6B).

**Figure 7:**
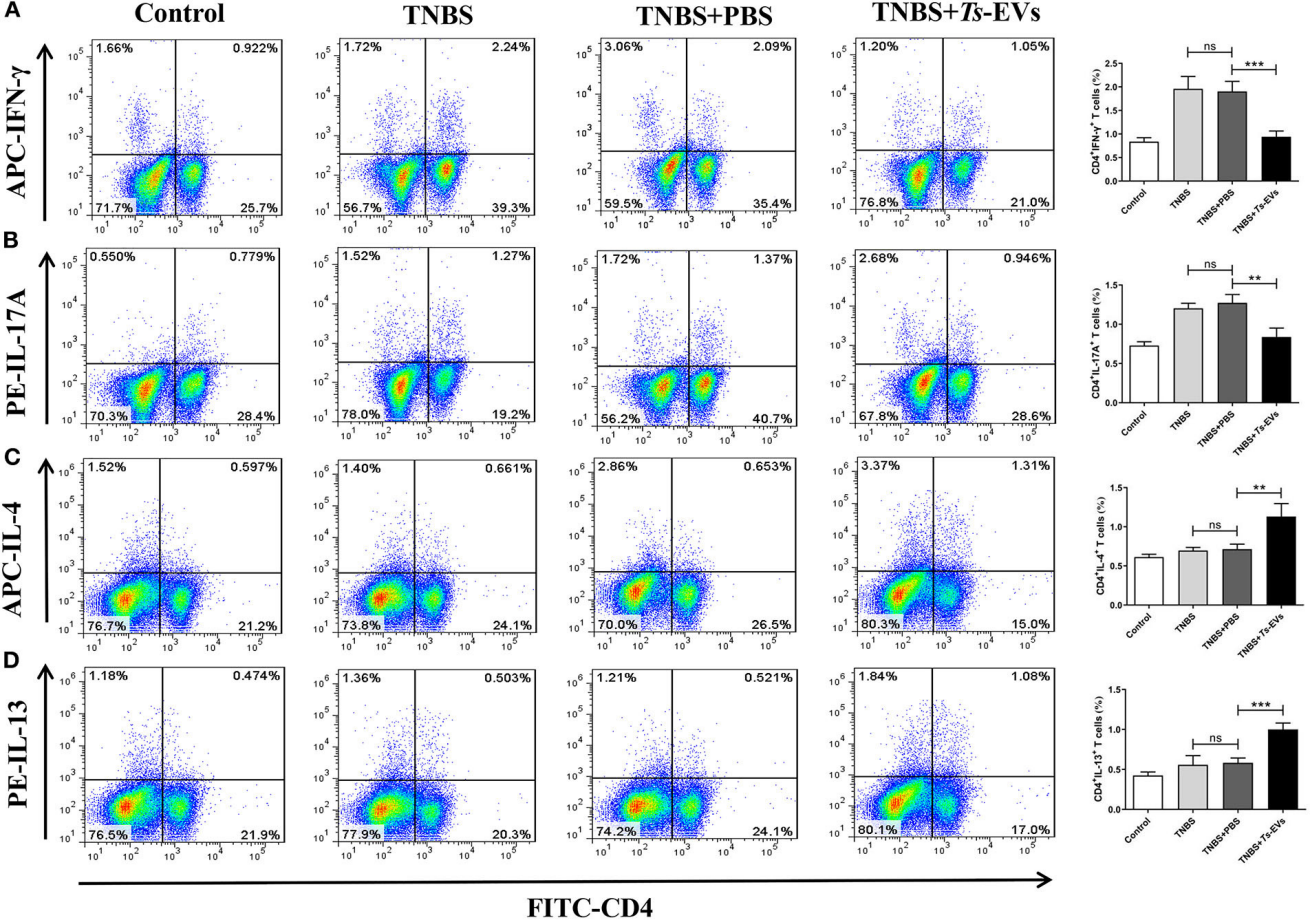
Flow cytometry analysis of Th1, Th2 and Th17 cells in the MLNs of mice with TNBS-induced colitis pretreated or not with *Ts*-EVs. Mesenteric lymph node (MLNs) cells were isolated from the different groups of mice, stimulated with Leukocyte Activation Cocktail containing PMA, ionomycin and GolgiPlug™ (Brefeldin A), and stained with specific antibodies. **(A)** Th1 (CD4^+^IFN-γ^+^) cells. **(B)** Th17 (CD4^+^IL-17A^+^) cells. **(C,D)**, Th2 (CD4^+^IL-4^+^ and IL-13^+^) cells. Results are shown as mean ± SD of each group (*n* = 3). Statistical analysis was performed with one-way ANOVA followed by Tukey's multiple-comparison test. ^**^p < 0.01, ^***^p < 0.001.

To further investigate the effects of *Ts*-EVs on Th cell differentiation, MLN cells were isolated from the different groups and analyzed by flow cytometry. This analysis showed upregulated populations of CD4^+^IFN-γ^+^ Th1 cells and CD4^+^IL-17A^+^ Th17 cells in the TNBS group when compared with the control group. In contrast, administration of *Ts*-EVs before colitis induction significantly decreased the populations of Th1 and Th17 cells when compared with the TNBS group (Figures 7A,B). Conversely, mice treated with *Ts*-EVs showed distinctly increased populations of CD4^+^IL-4^+^ Th2 cells and CD4^+^IL-13^+^ Th2 cells when compared with the TNBS group (Figures 7C,D). Thus, our results clearly indicate that the ability of *Ts*-EVs to ameliorate colitis is associated with an expansion of Th2 cells and a reduction of Th1 and Th17 cells in the MLNs.

**Figure 8:**
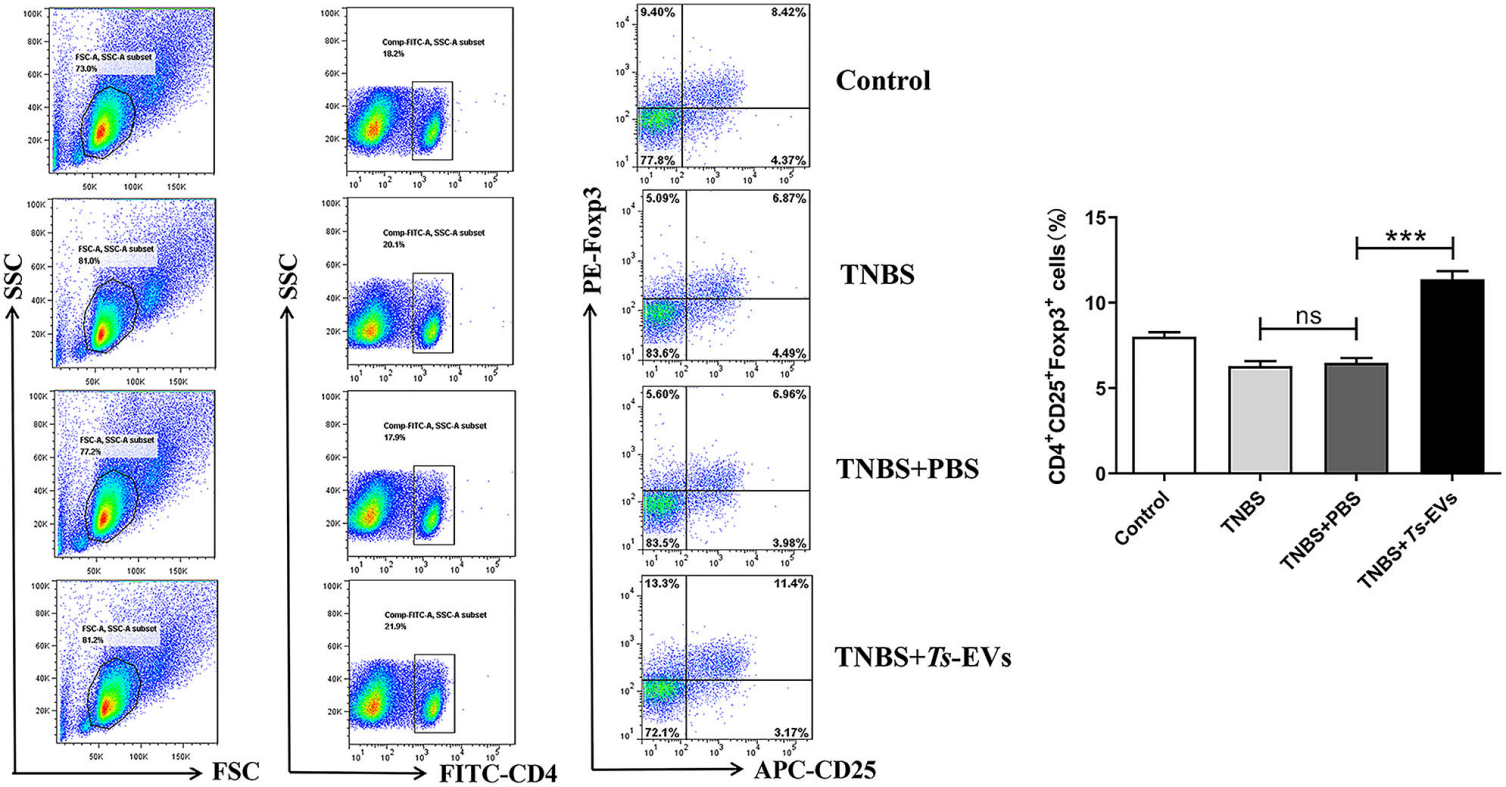
Flow cytometry analysis of Treg cells in the MLNs of mice with TNBS-induced colitis pretreated or not with *Ts*-EVs. The percentages of CD4^+^CD25^+^Foxp3^+^ Treg cells are shown. Results are shown as mean ± SD of each group (*n* = 3). Statistical analysis was performed with one-way ANOVA followed by Tukey's multiple-comparison test. ^***^p < 0.001.

CD4^+^CD25^+^Foxp3^+^ Treg cells play a pivotal role in maintaining immune tolerance and reducing the intensity of inflammation. Foxp3^+^ is one of the key transcription factors that control the development and function of Treg cells and plays a key role in regulating Treg activity. To measure changes in the levels of Tregs in mice with TNBS-induced colitis which had been pretreated with *Ts*-EVs, we isolated cells from the MLNs. As shown in Figure 8, we found a significant reduction in the percentage of CD4^+^CD25^+^ Foxp3^+^ Treg cells in the TNBS group, when compared with the control group. In contrast, mice pretreated with *Ts*-EVs had a reproducible and marked increase in the percentage of CD4^+^CD25^+^Foxp3^+^ Treg cells (Figure 8). Hence, our data indicate that *Ts*-EVs exert a protective effect against TNBS-induced acute colitis by upregulating Treg cells.

### *Ts*-EVs Contain Helminth-Specific miRNAs With Immunomodulatory Potential

**Figure 9:**
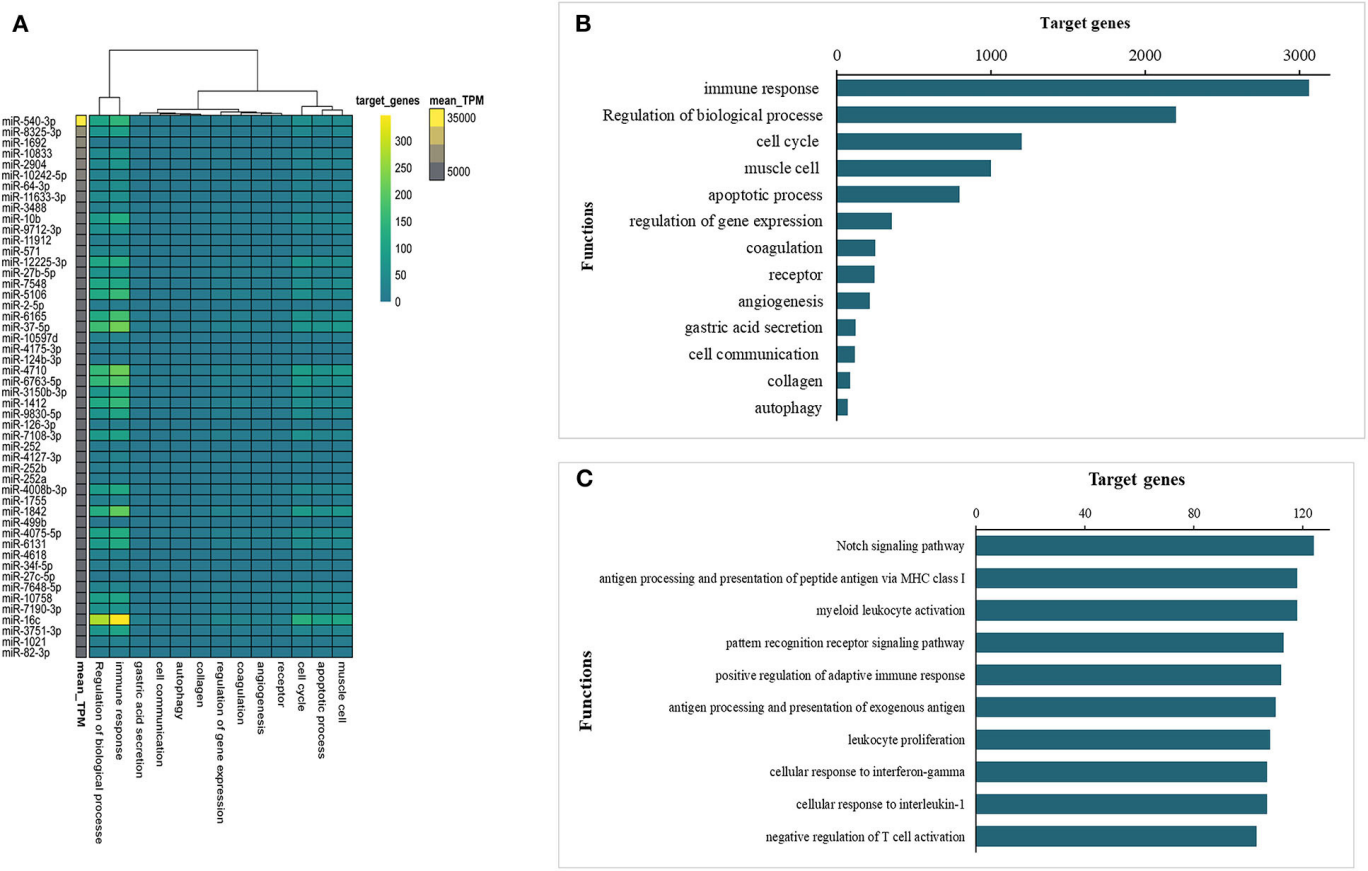
Prediction of interactions between *Ts*-EV miRNAs and murine host genes. **(A)** Functional map of *Ts*-EVs miRNAs and murine host target genes involved in various biological processes (heat map corresponds to individual target genes in the murine host based on the 50 most abundant miRNAs). Data are available in Table S1. **(B)** Total number of targeted gene networks identified and classified based on different biological process. Data are available in Table S1. **(C)** Total number of targeted gene networks identified and classified as “immune response.” Data are available in Table S1.

miRNAs contained in EVs play an important role in EV-mediated host-parasite communication. Therefore, the miRNA content of *Ts*-EVs was characterized using the Illumina HiSeq platform together with bioinformatics analysis. The high throughput sequencing results showed that the length distribution of the miRNAs was 18–30 nt, with 25 nt as the main length. Based on the abundance of transcripts per million (TPM), the top 50 miRNAs with predicted important regulatory roles in various biological processes are shown in Figure 9. Our results show that the murine host genes targeted by the identified miRNAs are related to the immune response, angiogenesis, apoptotic process, autophagy, cell communication, cell cycle, coagulation and other biological processes (Figures 9A,B). Interestingly, the immune response-associated target genes are mostly involved in antigen processing and presentation, pattern recognition receptor signaling pathways, cellular response to cytokines IL-1 and INF-γ, regulation of the adaptive immune response and leukocyte activation (Figure 9C).

## Discussion

The growth, development and long-term survival of *T. spiralis* in the host depend on biologically active, immunoregulatory compounds released by the parasite. These allow *T. spiralis* to create an immunologically friendly environment for successfully parasitism. In addition, they can also ameliorate autoimmune diseases caused by exacerbated immune responses, such as IBD (15). In recent years, extracellular vesicles (EVs) released by parasites have been the focus of extensive interest as “bridges” communicating parasites with the host during infection (16).

In this study, we isolated EVs from *T. spiralis* muscle larvae and evaluated their immunoregulatory potential in the TNBS-induced murine colitis model. First, we used TEM and NTA to identify the morphology and physical properties of the isolated EVs. The EVs were closed circular or elliptical vesicular structures ranging in size from 50 to 250 nm, with an average diameter of 178 nm. The size and characteristics of the EVs we isolated were similar to those described in a previous study and also to those released by other helminths, such as *Schistoso*ma mansoni and *Trichuris muris* (17, 18). The transmembrane protein CD63 and enolase are common surface markers of extracellular vesicles in many cell types and are usually used to identify EVs after isolation (19, 20). Our Western blot results showed high expression of CD63 and enolase in *T. spiralis* EVs. Collectively, our results confirmed the high quality of the *Ts*-EVs we obtained and established that the isolated EVs could be used in subsequent experiments.

The mechanisms by which parasite-secreted molecules restrain host immune responses has been investigated in various mouse models of disease, with roles ascribed to expansion of immunoregulatory cells like tolerogenic dendritic cells, alternatively activated macrophages and regulatory T and B cells, as well as suppression of inflammatory T helper (Th)-1 and−17 responses and induction of parasite-specific Th2 responses (21, 22). In this study, we evaluated whether *Ts*-EVs could ameliorate the development of TNBS-induced experimental colitis in mice by intraperitoneal injection of *Ts*-EVs, and explored the underlying mechanisms. Our results showed that pretreatment of mice with *Ts*-EVs reduced clinical signs of colitis as determined by the DAI score, inflammatory cell infiltration and intestinal histopathology. Different injection routes of EVs have been proved to influence their tissue distribution, and transperitoneal injections resulted in significantly higher EV accumulation in gastrointestinal tract (23). Helminth-derived EVs were also previously found to be capable of internalizing by murine small intestinal organoids, and transperitoneal injection of parasite-derived EVs could attenuate mucosal intestinal damage and prevent intestinal inflammation in experimental models (10). Accordingly, we supposed, the *Ts*-EVs could enrich in the gastrointestinal tract after IP injection, and then might be taken up by different host immune cells, and further modulated intestinal immunity through various mechanisms. It was quite unfortunate that, in our present study, the distribution of the *Ts*-EVs in the colon of mice and the mechanisms of EVs with protein and miRNA cargo internalization by host cells, such as macrophages, DC and intestinal epithelial cell were not explored, but will be further studied.

It is well-known that pro-inflammatory cytokines play a pivotal role in the initiation and amplification of inflammation by recruiting and activating leucocytes (24). Improvement of inflammatory disorders by infection with *T. spiralis* and other parasites has been ascribed to the anti-inflammatory properties of their secreted molecules (25, 26). We examined whether *Ts*-EVs could protect against IBD by exerting anti-inflammatory effects. We found that *Ts*-EVs dramatically reduced the expression of pro-inflammatory cytokines, whereas the levels of anti-inflammatory cytokines like IL-10 and TGF-β increased significantly in colon tissue of mice with TNBS-induced colitis. These results are similar to those reported in previous studies for EVs isolated from *Nippostrongylus brasiliensis* and *Fasciola hepatica*, which reduced the severity of inflammation in a murine colitis model (10). Our results indicate that *Ts*-EVs have the ability of modulating the balance between pro-inflammatory and anti-inflammatory cytokines, which helps improve the intestinal histopathology.

Chemokines are a family of structurally related cytokines which play a fundamental role in the regulation of leukocyte recruitment and activation in the colon (27). In the present study, we found that pretreatment with *Ts*-EV*s* decreased the expression of macrophage inflammatory protein-3α (MIP-3α) in the intestines of mice with colitis. MIP-3α induces CD4^+^ T and immature dendritic cell transmigration and plays an important role in colonic adaptive immune responses. Therapy with anti-MIP-3α mAbs significantly ameliorated colonic injury and reduced intestinal inflammation induced by TNBS in mice (28). We hypothesize that the decreased levels of MIP-3α in the colons of mice treated with *Ts*-EVs reduce the influx of CD4^+^T cells and dendritic cells, which in turn improves colonic inflammation. Further studies are needed to clarify what other chemokines are regulated by *Ts*-EVs.

MPO is mainly present in the cytoplasm of neutrophils, and its activity reflects the degree of infiltration by neutrophil inflammatory cells in damaged tissues. Therefore, MPO can be used to evaluate disease severity in colitis (29). Our results showed that MPO activity in the TNBS+*Ts*-EVs group was significantly reduced, indicating that inflammatory cell infiltration in colonic tissue was significantly diminished. Intestinal barrier dysfunction plays a pivotal role in the initiation and acceleration of mucosal inflammation, and is an important pathogenic factor in the development of IBD. Tight junction proteins play a key role in maintaining the intestinal barrier. Pro-inflammatory cytokines, such as IFN-γ and TNF-α, induce instability of epithelial TJ proteins involved in maintaining intestinal epithelial integrity, leading to epithelial barrier dysfunction (30). Anti-inflammatory agents can improve intestinal barrier function. Occludin and ZO-1 are key members of the tight junction protein family and play an important role in maintaining the integrity of the intestinal mucosal barrier (31). Interestingly, administration of *Ts*-EVs increased occludin and ZO-1 expression and protected against epithelial junction damage in the TNBS-induced colitis model, thereby contributing to maintain the integrity of the intestinal barrier. Moreover, we investigated the influence of *Ts*-EVs on intestinal permeability. The result showed that *Ts*-EVs restored the impaired permeability induced by TNBS in mice, which further indicated that *Ts*-EVs could improve the intestinal integrity damaged by inflammation. These results highly confirmed that *Ts*-EVs exert potent anti-inflammatory effects.

Aberrant activation and differentiation of Th1 cells which produce interferon-γ (IFN-γ) and Th17 cells which produce IL-17 is generally associated with autoimmune diseases, especially IBD. Th2-type and regulatory T cell responses and production of Th2/immunoregulatory cytokines, e.g., IL-4, IL-13, IL-10, and TGF-β play a crucial role in protecting against Th1/Th17 immune-mediated diseases. TNBS-induced colitis is characterized by transmural inflammation, a process that is regulated by Th1/Th17 immune responses, along with overproduction of interferon IFN-γ and IL-17 in the colonic mucosa (32). Thus, TNBS-induced colitis is a valuable model to investigate the regulation of T cell activation and differentiation by parasites and their products. To investigate *Ts*-EVs immunoregulatory mechanisms, we evaluated their ability to modulate the balance between different T cell subpopulations in this model. A recent study showed that EVs released by *Fasciola hepatica* exerted a protective effect in DSS-induced colitis that was independent of adaptive immunity (11). In our study, mice treated with *Ts*-EVs showed decreased expression of the Th1 cytokine IFN-γ and the Th17 cytokine IL-17A, and of their transcriptional factors T-bet and RORγt as well. Consistently, intracellular staining showed that the percentages of IFN-γ-producing CD4^+^ T cells and IL-17A-producing CD4^+^ T cells in the MLNs of *Ts*-EVs-treated mice were significantly lower than in the TNBS group. Based on these results, we believe that *Ts*-EVs are vigorous immune regulators with the capacity to strongly modulate the Th1/Th17 axes in the context of intestinal damage.

In addition to reduced Th1/Th17 immune responses, we also observed increased expression of Th2 (IL-4, IL-13, GAGT3) and Treg (IL-10, TGF-β and Foxp3)-associated cytokines and transcription factors in mice pretreated with *Ts*-EVs when compared with the TNBS group, and these results were confirmed by flow cytometry. These data suggest that the capacity of *Ts*-EVs to induce Th2/Treg cell differentiation also explains their anti-inflammatory effects. Similar results have been reported in *T. spiralis*, since specific antigens of this parasite, like serine protease inhibitors, can promote Th2 or /and Treg immune responses (33). DCs are the key link between innate and adaptive immunity, and play a critical role in the activation and differentiation of T lymphocytes. Thus, they could be a key target for regulating the host immune response by parasites. DCs interact with ES products and parasite molecules which can induce a tolerogenic immune microenvironment by regulating the balance between T cell subgroups, ultimately impacting autoimmune disease progression in mouse models (34, 35). For example, mice with experimental autoimmune encephalomyelitis (EAE) injected with DCs treated with *Ts*-MLES showed significantly reduced EAE severity, an outcome associated with downregulation of Th1/Th17 responses, boosting of regulatory T cells and increased secretion of regulatory IL-10 and TGF-β cytokines (36). In our previous study, we found that adoptive transfer of *Ts*-MLES-DC alleviated disease activity in mice with TNBS-induced colitis by shifting the immune response from Th1 toward Th2 and regulatory (37). Recent studies indicated that extracellular vesicles from the *Echinococcus granulosus* larval stage were internalized by dendritic cells and interfered with their antigen presentation function by downregulating MHCII expression (8). It would be interesting to investigate the potential of *Ts*-EVs to regulate DC maturation and subsequent T cell polarization.

MicroRNAs (miRNAs) are small non-coding RNA molecules that are emerging as important post-transcriptional inhibitors. They negatively regulate target gene expression by degrading mRNA or repressing translation. A growing body of evidence indicates that miRNAs are involved in the regulation of numerous biological processes, such as cell development, differentiation, proliferation, apoptosis, and in various diseases (38). There are also numerous studies showing that miRNAs regulate a wide spectrum of immune system functions and shape both innate and adaptive immunity, including Th1 and Th2 polarization and inflammatory responses (39, 40). Some studies have also found that pathogen-derived microRNAs can regulate host immune responses via cross-species interactions, subverting the immune system during infection (41). Investigations of extracellular vesicles released by parasitic helminths showed they contain abundant miRNAs and that these exosome-derived miRNAs participate in parasite-driven immunoregulation. Recent studies found that *S. japonicum* miRNAs can be delivered to host macrophages and T helper cells, where they regulate macrophage proliferation and Th cells differentiation by altering target gene expression (42, 43). In line with these studies, prediction of the interactions between microRNAs from *Ts*-EVs and murine host genes indicated that these microRNAs may play a vital role in the modulation of host immune system functions, such as antigen presentation and immune cell activation. Further research is necessary to investigate whether miRNAs from *T. spiralis* can be transported into host cells via *Ts*-EVs as an important mechanism to regulate host genes associated with intestinal immunity and inflammation. However, in depth elucidation of how *Ts*-EVs miRNAs subvert the host immune defense system will provide novel insights into *T. spiralis*-host interactions.

In conclusion, *Ts*-EVs showed immunoregulatory effects in a mouse model of IBD. The protective effects of *Ts*-EVs against TNBS-induced colitis involved regulation of Th1/Th2 balance and induction of Treg differentiation. These effects partly reproduced the beneficial effects of *T. spiralis* self and ES products. miRNAs contained within the EVs could be assigned to target genes which regulate the host immune response. These findings expand our knowledge of the mechanisms by which *T. spiralis* evades the host immune system and suggest new therapeutic approaches for IBD and other autoimmune disorders. However, we did not identify the specific miRNAs or proteins in *Ts*-EVs which mediate the immunoregulatory effects. Further hotspot studies are needed to identify the specific cargo molecules carried by *Ts*-EVs and to clarify their function during *T. spiralis* infection.

## Data Availability Statement

The datasets generated for the study can be found in NCBI Sequence Read Archive (SRA) under accession code PRJNA631364.

## Ethics Statement

The animal study was reviewed and approved by Ethics Committee of Jilin University, affiliated with the Provincial Animal Health Committee, Jilin Province, China.

## Author Contributions

The study was conceived and designed by XB and ML. YY, LL, and YZ performed the experiments. YY, XB, and XL analyzed the data. YY, LL and XB wrote the manuscript. XL, HS, WJ, HZ, HJ, and ML improved the manuscript. All authors read and approved the final manuscript.

## Conflict of Interest

The authors declare that the research was conducted in the absence of any commercial or financial relationships that could be construed as a potential conflict of interest.

## Supplementary Material

Table S1 Prediction of *Ts*-EV miRNA interactions with murine host target genes. Heat map corresponds to individual target genes in the murine host based on the 50 most abundant miRNAs by R package pheatmap. Functional map of *Ts*-EV miRNAs and murine host target genes categorized by ggplot2 package.
